# Clinical manifestations of 17 Chinese children with hereditary spherocytosis caused by novel mutations of the *ANK1* gene and phenotypic analysis

**DOI:** 10.3389/fgene.2023.1088985

**Published:** 2023-02-01

**Authors:** Meiyun Kang, Huimin Li, Jun Zhu, Liwen Zhu, Yue Hong, Yongjun Fang

**Affiliations:** ^1^ Department of Hematology and Oncology, Children’s Hospital of Nanjing Medical University, Nanjing, China; ^2^ Key Laboratory of Hematology, Nanjing Medical University, Nanjing, China

**Keywords:** hereditary spherocytosis, ANK1, mutation spectrum, genotype, phenotype

## Abstract

**Background:** Hereditary spherocytosis (HS) is an autosomal dominant (AD) and autosomal recessive (AR) disorder that is mostly caused by mutations of the erythrocyte membrane-related gene *ANK1*.

**Methods:** Clinical and genetic testing data of 17 HS children with *ANK1* gene mutations were retrospectively collected. Clinical manifestations and phenotypic analysis of HS were summarized based on our experience and literature review.

**Results:** A total of 17 mutations of the *ANK1* gene were identified from 17 probands (12 sporadic cases and five familial cases), including 15 novel mutations and two previously reported ones. Among the 15 novel variants of *ANK1*, there were four non-sense mutations, four frameshift mutations, three splicing mutations, three missense mutations and one in-frame deletion of three amino acids. In the present study, HS patients with mutations in membrane binding domains had significantly lower hemoglobin (Hb) levels and higher total bilirubin (T-Bil) levels than those with mutations in regulatory domains. After reviewing and analyzing all available published reports of Chinese HS patients carrying ANK1 mutations in PubMed and Chinese journals, there were no significant differences in Hb, Ret and T-Bil between different mutation types or mutation regions.

**Conclusion:** Mutations of the *ANK1* can be inherited or *de novo*. Clinical manifestations of HS in children caused by *ANK1* mutations are similar to those of other types of hemolytic anemia. Our report expands the mutation spectrum of HS, thus providing references for clinical management and genetic counseling of HS.

## Introduction

Hereditary spherocytosis (HS) is a type of heterogeneous disorder that belongs to the common form of congenital hemolytic anemia (HA). It is characterized by the presence of sphere-shaped red blood cells (spherocytes) on the peripheral blood smear, anemia, jaundice and splenomegaly. HS is featured by a huge heterogeneity in disease severity among patients, whom may be virtually asymptomatic or require transfusions frequently in early childhood. Therefore, the diagnosis of HS is challenging, particularly for asymptomatic or atypical cases that only depend on clinical manifestations, family history and hematologic laboratory testing (Bolton-Maggs et al., 2012). Through literature review, the incidence of HS is 1/2,000 among individuals of northern European ancestry (Perrotta et al., 2008). To date, accurate epidemiological data in China is scant. Wang et al. (2015) estimated in 2015 that the prevalence of HS in Chinese population was 1.27/1,00,000 in males and 1.49/1,00,000 in females. Since HS is generally an inherited disorder caused by gene mutations, gene mutation spectrum responsible for the pathogenesis of HS is of great significance. Conventional laboratory testing, however, often fails to diagnose HS. With the advanced medical technologies, molecular genetic testing, especially next-generation sequencing (NGS), is becoming a powerful tool to the clinical diagnosis of HS in neonates or infants through accurately identifying genetic variants (Christensen et al., 2015).

HS is attributed to gene mutations in erythrocyte membrane proteins (*ANK1*, *SPTA1*, *SPTB*, *SLC4A1*, and *EPB42*), which is inherited in the autosomal dominant (AD) or autosomal recessive (AR) manner (Perrotta et al., 2008). Their mutations usually result in the decreased membrane surface area relative to the intracellular volume of erythrocytes or dysfunction of the erythrocyte membrane, leading to the detachment of the lipid bilayer from the spectrin-based cytoskeleton. As a result, abnormal erythrocytes transform into spherocytes with an increased osmotic fragility, which are prone to be trapped and destroyed in the spleen. Consequently, hemolytic anemia and increased erythropoiesis lead to reticulocytosis, hyperbilirubinemia, gallstones, and splenomegaly (Bolton-Maggs et al., 2004). Thus, the definitive diagnosis of HS relies on molecular testing. Among the five virulence genes, the majority of HS cases are caused by mutations of the *ANK1* (He et al., 2018).

The *ANK1* gene is located at 8p11.21 and consists of 42 exons. It encodes the ankyrin-1 protein of 1,881 amino acids with three main domains, including a N-terminal membrane-binding domain containing binding sites for the band 3 protein, a central spectrin-binding domain involving two ZU5 domains and one UPA domain for interacting with the actin-spectrin cytoskeleton, and a C-terminal regulatory domain responsible for modulating the affinity of the other domains (Lux et al., 1990). Mutations have been identified throughout the entire gene, resulting in the lack of one haploid set of ankyrin-1 (Delaunay, 2002). HS caused by mutations of the *ANK1* is mainly inherited in the AD manner, with the most common types of non-sense, frameshift and splicing mutations. Most of patients with recessive HS are caused by mutations in the gene promoter and missense mutations. The frequency of spontaneous mutations of the *ANK1* is high, and approximately 15%–20% of reported mutations of the *ANK1* are *de novo* (Gallagher, 2005; Perrotta et al., 2008).

Genetic testing is recently popular in assisting the diagnosis of HS in many countries and regions, including China (Nakanishi et al., 2001; Wang et al., 2018). Wang et al. (2018) and Wu et al. (2021) have preliminarily expanded the mutation spectrum of genes responsible for HS, and clarified the mutational characteristics of causative genes, which should be further comprehensively elucidated.

In this case report, 17 children with anemia or spherocytosis caused by mutations of the *ANK1* were retrospectively analyzed to explore the clinical and mutational features of HS patients with mutations of the *ANK1*, thus providing references for clinical treatment and genetic counseling.

## Materials and methods

### Subjects

Children aged 0–14 years who were manifested with anemia or spherocytosis symptoms caused by mutations of the *ANK1* confirmed in the Department of Hematology and Oncology, Children’s Hospital of Nanjing Medical University, China from January 2018 to December 2021 were retrospectively recruited. All probands were from unrelated families, and they were clinically diagnosed as HS according to the guidelines by experienced hematologists (Bolton-Maggs et al., 2012). Their clinical data were retrospectively collected, including data of splenomegaly, jaundice, anemia, or cholecystolithiasis, therapeutic strategies and family history. Laboratory testing data, including blood cell counts, blood biochemical indexes and direct antiglobulin test before blood transfusion or splenectomy were collected as well.

After obtaining informed consent for clinical and genetic investigations, peripheral blood samples were collected from all patients and their parents. The study protocol was approved by the Ethics Committee of Children’s Hospital of Nanjing Medical University.

### DNA preparation

Peripheral blood samples (2 mL) were collected and placed in disposable vacuum tubes for genetic analysis. Genomic DNA was extracted from peripheral leukocytes using the DNA isolation kit (Tiangen, Beijing, China) according to the manufacturer’s protocol.

### Next-generation sequencing (NGS) and DNA sequencing analysis

DNA fragments in targeted regions were enriched using DNA microarrays and then sequenced on a high-throughput second-generation sequencing platform using the GenCap kit (MyGenostics GenCap Enrichment technology). The amplified DNA was captured by whole-exome sequencing (WES) of the exons of *ANK1* gene and its flanking untranslation regions (UTRs). The obtained sequences were aligned to the reference human genome (hg19 build) using BWA software. Single nucleotide variation (SNV), inserts and deletions (INDEL) were filtered by GATK software (https://software.broadinstitute.org/gatk/). All variants were further annotated by ANNOVAR software. The variant sites with frequency less than 0.05% in the public databases, including the Genome Aggregation Database (gnomAD), dbSNP, 1000 Genomes MAF (Chinese), ExAC and an in-house MAF database, were removed. Missense variants were then predicted by SIFT (http://sift.bii.a-star.edu.sg), PolyPhen-2 (http://genetics.bwh.harvard.edu/pph2/), MutationTaster and GERP++ for pathogenic forecasts and conservative projections, while splice sites were predicted by SPIDEX (http://www.deepgenomics.com/spidex). All candidate variants were clarified in accordance with the American College of Medical Genetics and Genomics (ACMG) criteria 22 and further validated by Sanger sequencing.

### Review of published Chinese HS patients with ANK1

The terms “ANK1,” “Chinese,” and “variants” or “mutations” were used to search for articles reporting on HS in PubMed and Chinese journals. ANK1 variants are indicated on the longest isoform (complementary DNA: NM_000037.4; protein: NP_000028.3), according to Human Genome Variation Society (HGVS) guidelines (www.hgvs.org/mutnomen).

### Genotype-phenotype correlation

The data considered for the genotype-phenotype correlation in the probands were: red blood cell count (RBC), Hb, MCV, MCH, MCHC, red cell distribution width (RDW-SD), red cell distribution width—coefficient of variation (RDW-CV), hematocrit (HCT), Ret%, T-Bil, and D-Bil. Genotypes were subdivided by different types of mutations (non-sense, frameshift, splicing, and missense) and the location of the mutations (membrane binding domain, spectrin binding domain, and regulatory domain).

### Statistical analysis

Statistical analyses were performed using the GraphPad prism 9 software (GraphPad Software, Inc., San Diego, CA, United States). Differences between groups were compared by the Kruskal–Wallis H test or Chi-square test. Two-tailed *p*-value < 0.05 was considered as statistically significant.

## Results

### Genetic analysis of HS

A total of 17 variants in exon 1, 2, 9, 14, 17, 19, 22, 23, 30, 31, 37, 38, and 39 of *ANK1* were detected in 17 probands (Table 1). The distribution of each variant in the *ANK1* gene was shown in Figure 2A. Among them, 15 variants were novel (P1-P15), including four non-sense mutations (c.2164C>T/p.Q722X, c.5299G>T/p.E1767X, c.4414C>T/p.Q1472X, c.3600C>A/p.C1200X), four frameshift mutations (c.2393_2394insTAGT/p.D800Qfs*1, c.1564delC/p.Q522Afs*12, c.5163_5173del/p.W1721Cfs*16, c.4399_4400insGA/p. Q1467Sfs*16), three splicing errors (c.2462-2A>G, c.1801-2A>G, c.3533-1G>A), three missense mutations (Figure 2B, c.2461G>A/p.G821R, c.4648A>G/p. I1550V, c.872T>G/p.L291R) and one in-frame deletion of three amino acids (c.52_54delTTC/p.F18del). Two mutations in P16 and P17 have been reported previously (Eber et al., 1996; Nakanishi et al., 2001; Xiong et al., 2015). The 17 variants were heterozygous except for that in P14, which was hemizygous.

**TABLE 1:** Mutations of the ANK1 gene identified in 17 patients with hereditary spherocytosis.

Patients	Amino acid	Protein	Location	Inheritance	Type/Effect	ACMG scoring
P1	c.2462-2A>G	—	Exon23	Father	Splicing	LP (PVS1 + PM2)
P2	c.2164C>T	p. Q722X	Exon19	Father	Non-sense	LP (PVS1 + PM2)
P3	c.2393_2394insTAGT	p. D800Qfs*1	Exon2	*De novo*	Frameshift	P (PVS + PS2 + PM2)
P4	c.1564delC	p. Q522Afs*12	Exon14	Mother	Frameshift	LP (PVS1 + PM2)
P5	c.1801-2A>G	—	Exon17	Mother	Splicing	LP (PVS1 + PM2)
P6	c.2461G>A	p. G821R	Exon22	*De novo*	Missense	LP (PS2 + PM2 + PP3 + BP1)
P7	c.5163_5173del	p. W1721Cfs*16	Exon39	Father	Frameshift	LP (PVS1 + PM2)
P8	c.4399_4400insGA	p. Q1467Sfs*16	Exon37	Father	Frameshift	LP (PVS1 + PM2)
P9	c.4648A>G	p. I1550V	Exon38	Mother	Missense	U (PM2 + BP4)
P10	c.5299G>T	p. E1767X	Exon39	*De novo*	Non-sense	LP (PS2 + PVS1_PM4 + PM2 + PP4)
P11	c.3533-1G>A	—	Exon30	Mother	Splicing	LP (PVS1 + PM2)
P12	c.52_54delTTC	p. F18del	Exon1	Father	In frame	U (PM2 + PM4)
P13	c.4414C>T	p. Q1472X	Exon37	Mother	Non-sense	P (PVS1 + PM2 + PP4)
P14	c.872T>G	p. L291R	Exon9	*De novo*	Missense	LP (PS2 + PM2 + PP3)
P15	c.3600C>A	p. C1200X	Exon30	Mother	Non-sense	LP (PVS1 + PM2)
P16	c.3754C>T	p. R1252X	Exon31	*De novo*	Non-sense	P (PVS1 + PS1 + PS2 + PM2)
P17	c.2390_2393del	p. L797Sfs*7	Exon22	*De novo*	Frameshift	P (PVS1 + PS1 + PS2 + PM2)

ACMG, the American College of Medical Genetics and Genomics; LP, likely pathogenic; P, pathogenic; U, uncertain significance; PVS, very strong; PS, strong; PM, moderate; PP, supportive.

**FIGURE 1:**
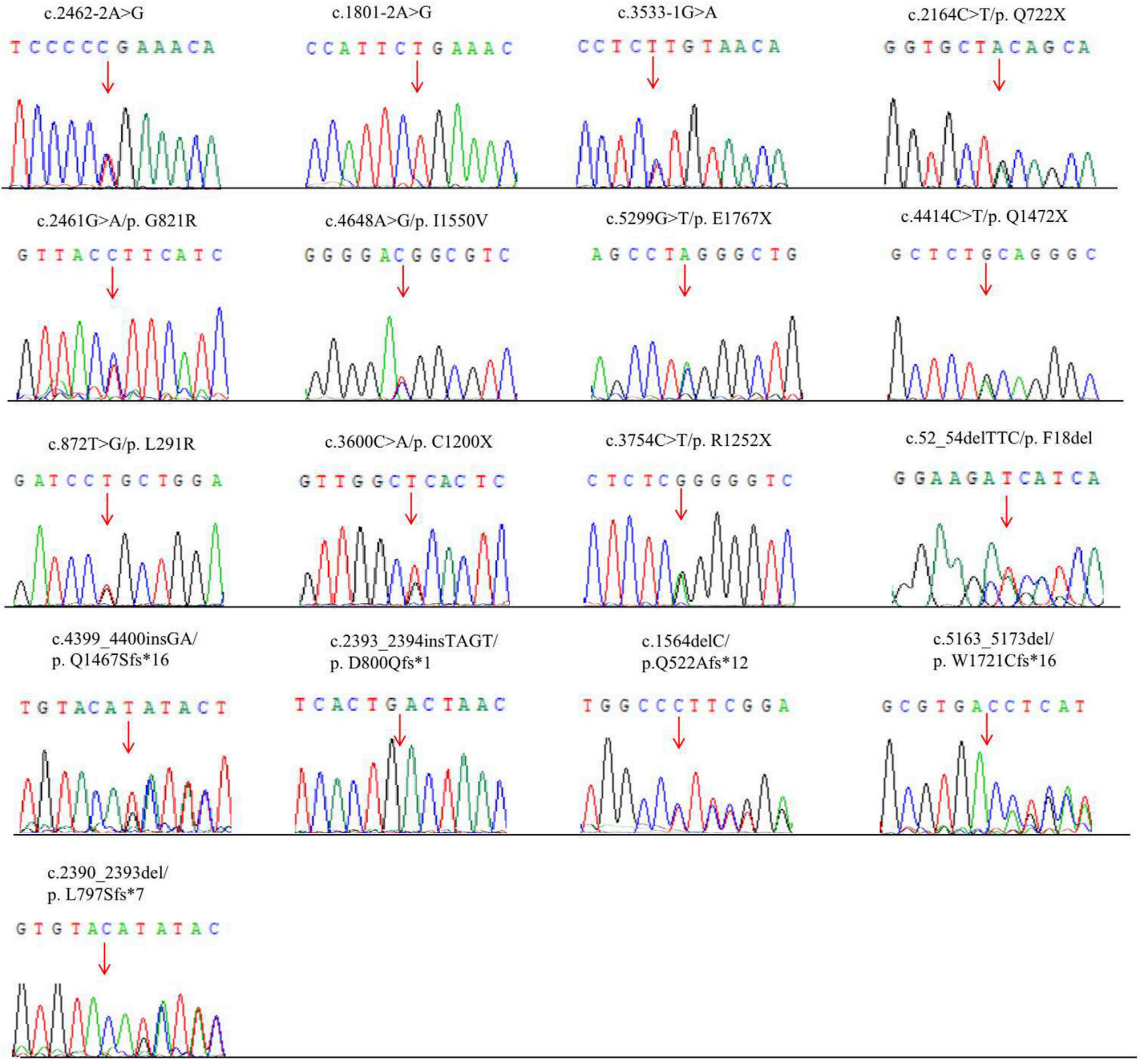
Novel mutations of the ANK1 gene identified in HS patients. Mutation sites are labeled by arrows.

Parental testing using Sanger sequencing on 15 HS patients revealed four *de novo* variants (c.2393_2394insTAGT/p. D800Qfs*1, c.2461G>A/p. G821R, c.5299G>T/p. E1767X and c.872T>G/p. L291R) (Figure 1). The remaining were inherited from their parents, with five inherited from their symptomatic father or mother.

**FIGURE 2:**
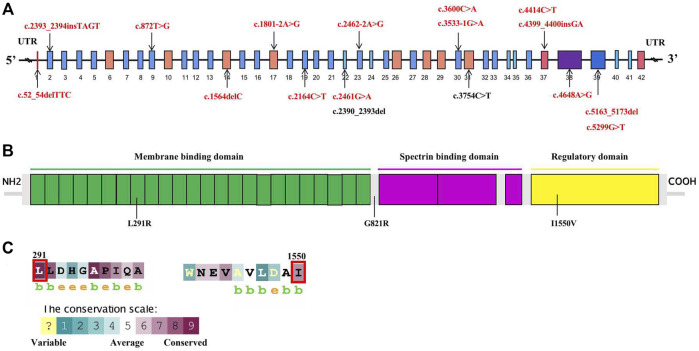
Schematic diagram of mutations of the ANK1 gene and conversation of amino acid residues affected by the missense mutations. **(A)** Mutations in the genome of ANK1. Fifteen novel mutations, and previously reported ones are highlighted in red bold and black bold, respectively. **(B)** The human erythroid ankyrin protein consists of an N-terminal membrane protein binding domain composed of ankyrin repeats (green box), a central spectrin-binding domain (violet box), and a C-terminal regulatory domain (yellow box). Three missense mutations are listed. **(C)** The evolutionary-conservation scores for residues encompassing the missense mutations in the ANK1 protein, reflecting the full alignment of 62 ANK1 homologues from different species, including mammals, lower vertebrates, invertebrates, and lower eukaryotes, as obtained from the UniRef90 database. The substituted residues, which are outlined by the red box in **(C)**, are highly conserved.

**FIGURE 3:**
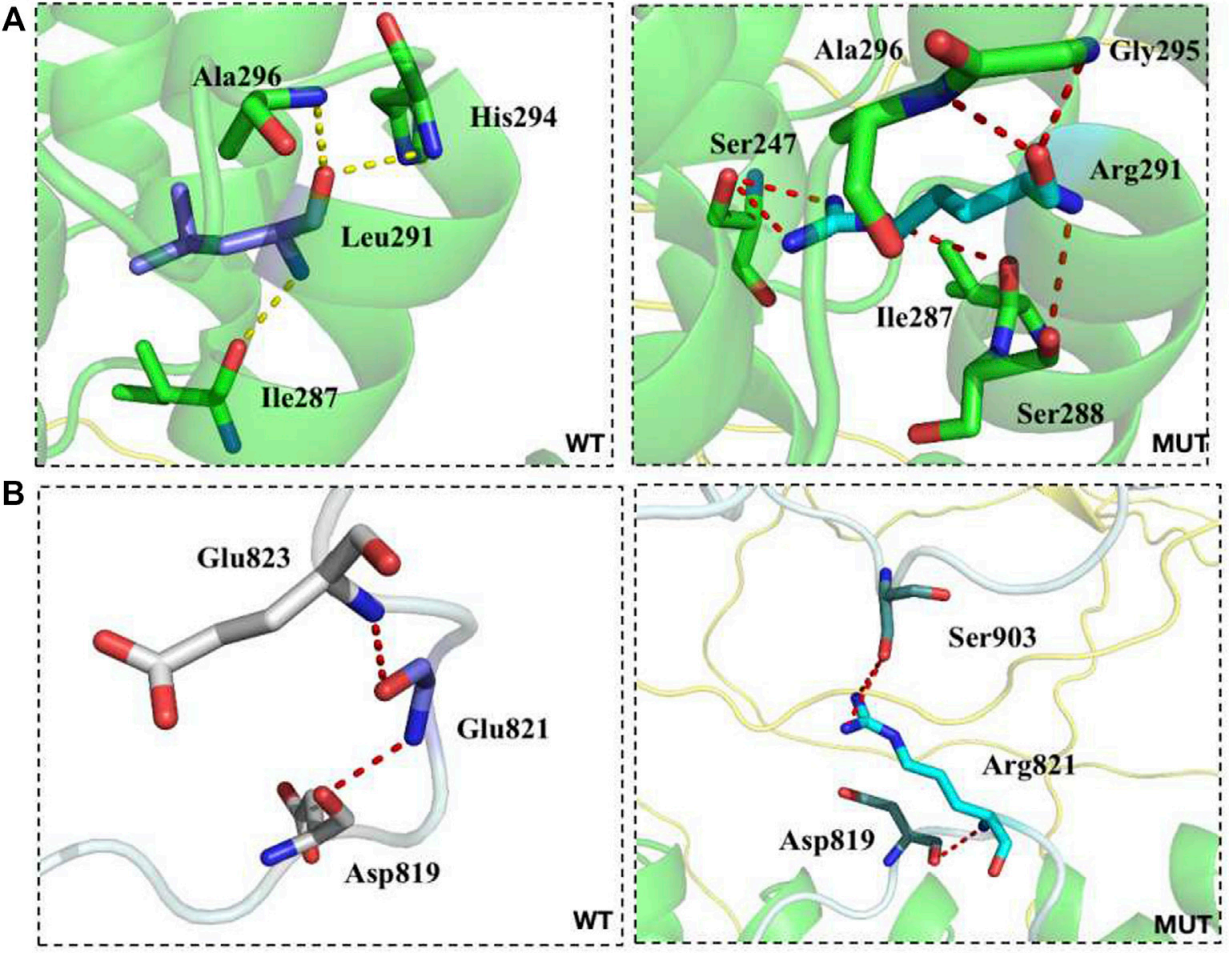
The transformation of amino acid hydrogen bonding force after mutation of L291R **(A)** and G821R **(B)**.

Conservative analysis of the three missense mutations was further performed, and their effect on protein conformation was predicted. It is shown that L291 and I1550 residues were evolutionarily conserved among 62 different species, including mammals, lower vertebrates, invertebrates, and lower eukaryotes (Figure 2C). We further performed simulated mutations on ANK1, and then summed up the effects (Figure 3). The transformation of amino acid hydrogen bond force after L291R mutation was shown in Figure 3A. Leu at position 291 was mutated to Arg, which broke hydrogen bonds with His at position 294 and formed hydrogen bonds with Gly at position 295, Ser at position 247 and Ser at position 288. Glu at position 821 was mutated to Arg after G821R mutation, which broke hydrogen bonds with Glu at position 823 and formed hydrogen bonds with Ser at position 903 (Figure 3B).

### Clinical manifestations and laboratory testing of HS

**FIGURE 4:**
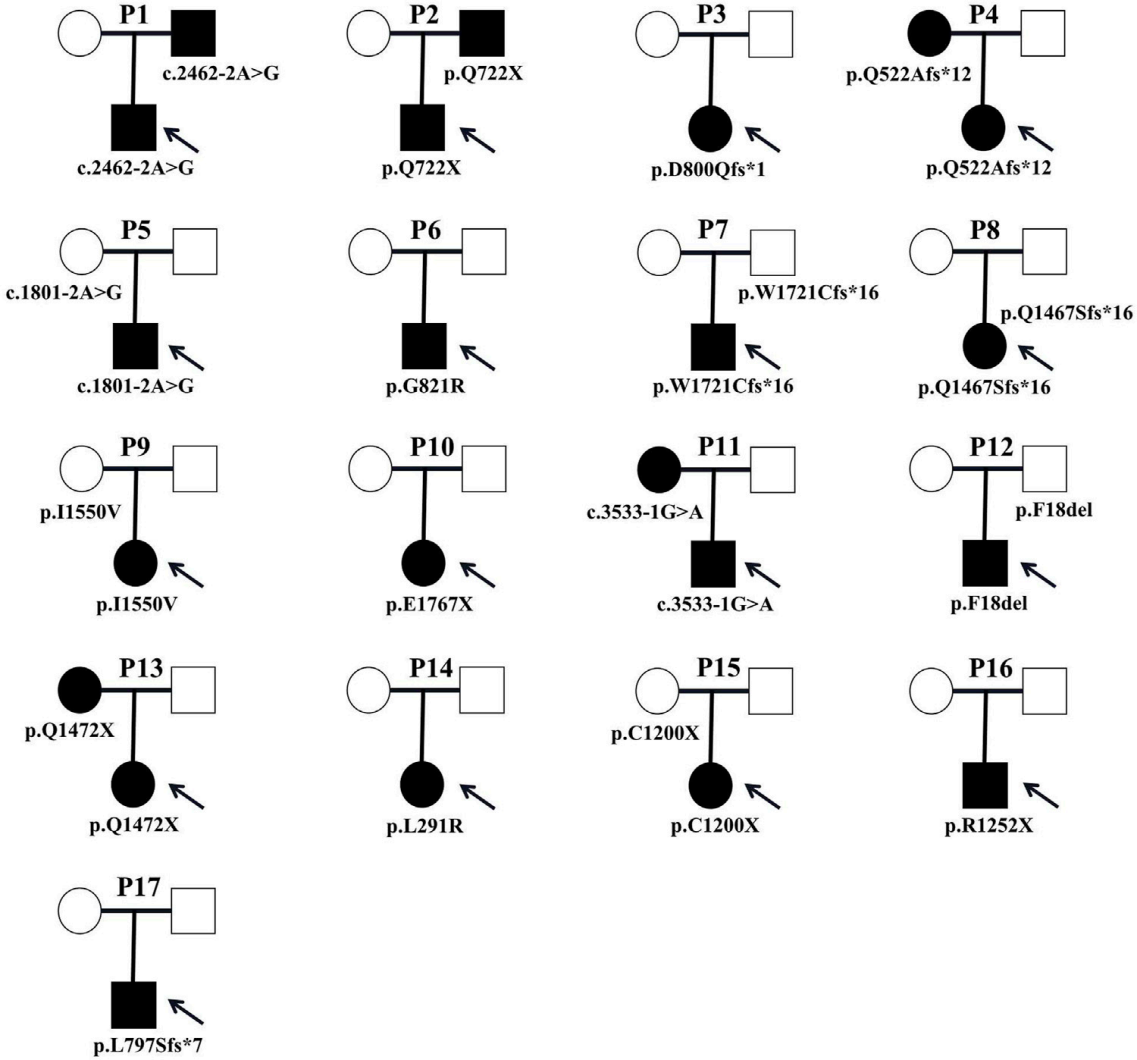
Pedigrees of affected families. Pedigrees of 17 families with ANK1 pathogenic variants. Probands are labeled by arrows. The proband and his father are labeled in the square, and the mother is labeled in the circle.

Clinical manifestations and laboratory testing data before splenectomy of the 17 probands were summarized in Table 2. All patients were unrelated, among whom 9 (52.9%) were male. The onset age ranged from 1 month to 13 years, with 4 (23.5%) had disease onset before 1 year of age. Almost all patients presented with jaundice, anemia and splenomegaly at varying severity. One patient (P3) developed the complication of cholecystolithiasis. Five patients (29.4%) had a family history of HS. The pedigrees of affected families were listed in Figure 4.

**TABLE 2:** Laboratory testing results of 17 patients with hereditary spherocytosis caused by *ANK1* mutations.

	P1	P2	P3	P4	P5	P6	P7	P8	P9	P10	P11	P12	P13	P14	P15	P16	P17
Gender	Male	Male	Female	Female	Male	Male	Male	Female	Female	Female	Male	Male	Female	Female	Female	Male	Male
Age	3 years	2 months	10 years	3 years	2 years	7 years	7 years	1 year	1 month	13 years	1 month	8 months	4 years	7 years	7 years	6 years	5 years
RBC (×10^12^/L)	2.82	2.06	3.28	2.24	2.84	2.76	3.95	2.41	3.1	3.71	1.8	4.45	3.34	2.93	2.05	3.54	2.14
Hb (g/L)	73.0	56.0	102.0	64.0	82.0	70.0	115.0	70.0	106.0	105.0	52.0	81.0	92.0	83.0	53.0	95.0	54.0
MCV (fL)	81.2	88.3	91.8	90.6	83.8	82.2	88.1	88.4	96.8	83.3	84.4	62.7	79.3	82.6	82.9	79.4	82.7
MCH (pg)	25.9	27.2	31.1	28.6	28.9	25.4	29.1	29	34.2	28.3	28.9	18.2	27.5	28.3	25.9	26.8	25.2
MCHC (g/L)	319.0	308.0	339.0	315.0	345.0	308.0	330.0	329.0	353.0	340.0	342.0	290.0	347.0	343.0	312.0	338.0	305.0
RDW-SD (fL)	63.7	66.1	74.1	85.9	61.2	73.4	54.4	83.6	55.2	63.1	53.2	39.4	55.8	61.1	60.4	63.3	94.2
RDW-CV (%)	22.7	22.8	23.1	28.6	20.3	28.6	16.9	26.5	15.6	21.4	17.7	17.2	20.5	21.3	24.9	22.7	35.1
HCT (fL)	22.9	18.2	30.1	20.3	23.8	22.7	34.8	21.3	30.0	30.9	15.2	27.9	26.5	24.2	17.0	28.1	17.7
Ret (%)	9.56	7.12	13.89	18.49	9.20	12.72	2.93	14.77	/	12.67	9.28	1.84	16.70	8.80	14.37	11.94	21.62
T-Bil (μmol/L)	174.80	55.70	86.46	/	53.20	85.80	37.38	24.30	/	46.90	29.30	/	34.10	/	57.10	33.15	81.10
D-Bil (μmol/L)	32.00	23.90	8.94	/	16.60	11.6	12.16	9.30	/	12.70	12.40	/	11.30	/	8.70	11.67	10.10
LDH (U/L)	396.0	447.0	317.0	/	331.0	313.0	248.0	551.0	/	274.0	324.0	/	541.0	/	468.0	319.0	417.0
Splenomegaly	+	+	+	NA	+	+	+	+	NA	+	+	-	+	-	+	+	+
Jaundice	+	+	+	NA	+	+	+	+	NA	+	+	NA	+	NA	+	+	+
Cholecystolithiasis	-	-	+	-	-	-	-	-	NA	-	-	-	-	-	-	-	-
Cholecystectomy	-	-	+	-	-	-	-	-	NA	-	-	-	-	-	-	-	-
Splenectomy	+	-	+	-	-	+	-	-	-	-	-	-	-	-	+	-	+
Family history	+	+	-	+	-	-	-	-	-	-	+	-	+	-	-	-	-
Blood transfusion	+	+	-	+	-	+	-	+	-	-	+	-	-	-	+	-	+

RBC, red blood cells, reference range: 3–10; Hb, hemoglobin, reference range: 120–156; MCV, mean corpuscular volume, reference range: 72–92; MCH, mean corpuscular hemoglobin, reference range: 24–34; MCHC, mean corpuscular hemoglobin concentration, reference range: 309–359; RDW-SD, standard deviation of red blood cell distribution width, reference range: 37–47; RDW-CV, coefficient of variation of red blood cell distribution width, reference range: 11.5–14.5; HCT, hematocrit, reference range: 30–47; Ret, reticulocyte, reference range: 0.5–1.5; T-Bil, total bilirubin, reference range: 3.4–17.1; D-Bil, direct bilirubin, reference range: 0–6.8; LDH, lactate dehydrogenase, reference range: 120–420.

NA, not available; +, positive; -, negative; /, not detected.

The mean Hb and reticulocyte ratio (Ret%) were 79.59 g/L and 11.62%, respectively. Most of HS patients had normal ranges of mean corpuscular volume (MCV), mean corpuscular hemoglobin (MCH) and mean corpuscular hemoglobin concentration (MCHC). T-Bil levels were elevated in all patients, with indirect bilirubin (I-Bil) predominated. The direct antiglobulin test was conducted in some HS patients to exclude autoimmune hemolytic anemia (ALHA), and all testing were negative. Eight patients (47.1%) were treated with blood transfusion due to severe anemia. Splenectomy was performed on five patients (29.4%), and P3 was intervened by splenectomy and cholecystectomy for cholecystolithiasis after a comprehensive assessment of risks and benefits. Symptoms of surgically treated HS patients improved significantly after surgery.

### Genotype-phenotype correlation in HS patients with mutations of the *ANK1*

**FIGURE 5:**
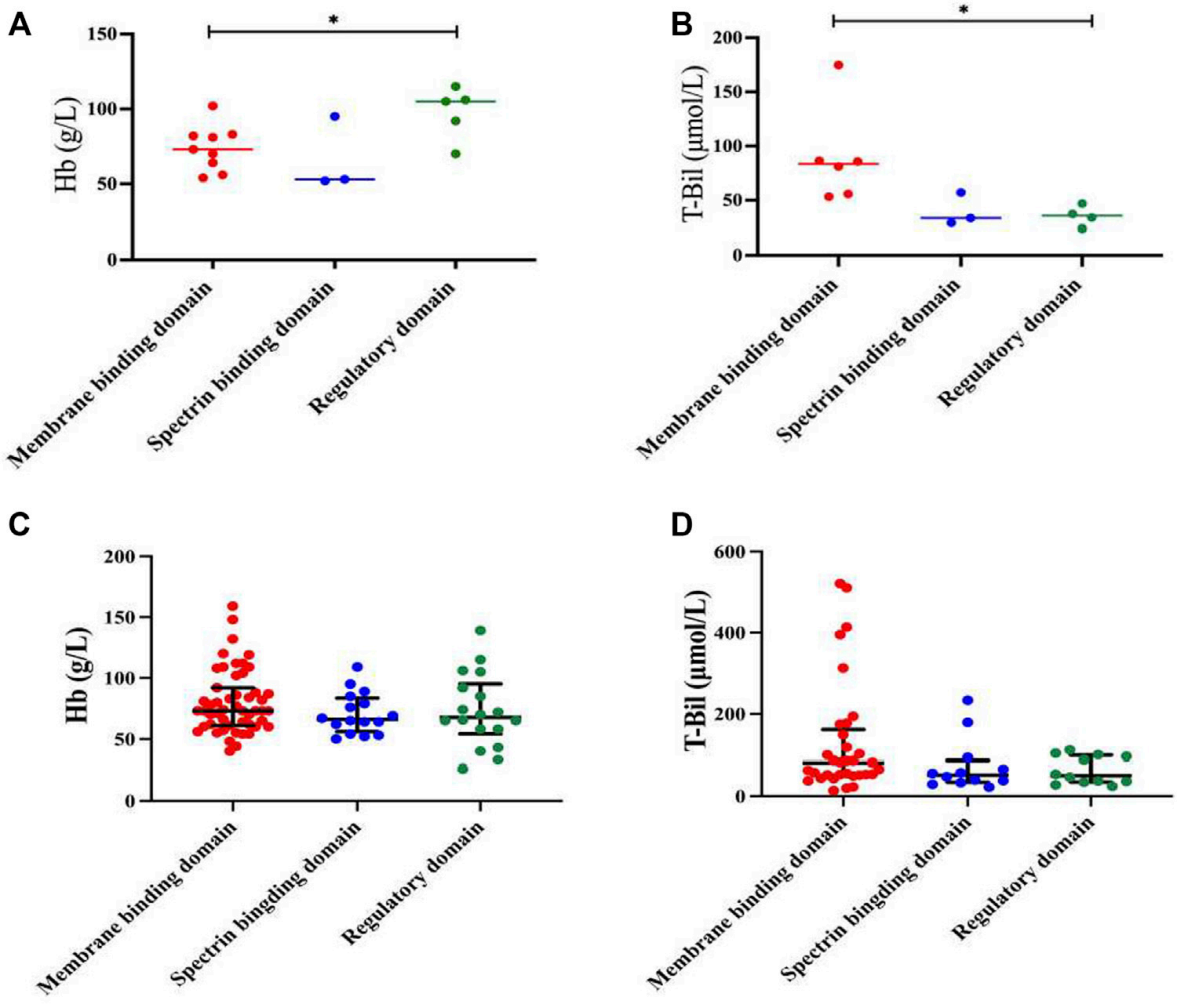
Genotype-phenotype correlation in HS patients with mutations of the ANK1. **(A,B)** Analysis of Hb and T-Bil between mutations of different locations (membrane binding domain, spectrin binding domain and regulatory domain) in 17 HS patients. **(C,D)** Analysis of Hb and T-Bil between mutations of different locations in all reported Chinese HS patients and our cases.

In our study, HS patients with mutations of the *ANK1* in regulatory domains had significantly higher Hb levels than those with mutations in membrane binding domains (*p* = 0.029, Figure 5A). In addition, T-Bil levels of HS patients with mutations of the *ANK1* in membrane binding domains was significantly higher than those with mutations in regulatory domains (*p* = 0.028, Figure 5B). Other clinical manifestations and laboratory testing data of HS patients carrying different mutation types and regions were shown in Supplementary Tables S1, S2, respectively.

After reviewing all available published reports of Chinese HS patients carrying ANK1 mutations, a total of 129 reported and 15 unreported ANK1 variants were further summarized, including missense (*n* = 19), frameshift (*n* = 40), non-sense (*n* = 46), and splicing (*n* = 21) variants (Supplementary Table S3) (Huang et al., 2019; Bin et al., 2020; Chai et al., 2020; Wang et al., 2020; Zhu et al., 2020; Wang et al., 2021a; Wu et al., 2021a; Xie et al., 2021a; Wang et al., 2021b; Xie et al., 2021b; Wang et al., 2021c; Fu et al., 2022; Li et al., 2022; Xiang and Shen, 2022; Zhao et al., 2022; Xu et al., 2023; Zhu et al., 2023). When we extended the Hb and T-Bil data to all 144 probands, the difference in Hb between regulatory domains and membrane binding domains disappeared (Figure 5C). T-Bil levels of HS patients with *ANK1* mutations in membrane binding domains were higher than those with mutations in spectrin binding domain and regulatory domains, although significant differences were not detectable (*p* = 0.129 and *p* = 0.164, respectively) (Figure 5D).

## Discussion

HS is a kind of non-immune hemolytic anemia, which is mainly inherited in an AD manner (Iolascon et al., 2019). Typical clinical manifestations of HS are similar to those of other types of hemolytic anemia. The severity of HS varies widely, and usually, HS occurs in infancy presents a serious phenotype. Laboratory testing, such as blood cell counts and erythrocyte osmotic fragility test (OFT), lacks ideal sensitivity or specificity in the diagnosis of HS (Wu et al., 2021b). Due to the challenge in timely diagnosis of HS by conventional examinations, misdiagnosis and missed diagnosis result in the poor prognosis and enhance the medical expenses of affected patients and their families. More seriously, undiagnosed HS may lead to complications like kernicterus, hemolytic episodes, aplastic crisis and cholecystolithiasis. In our study, the mean onset age of 17 HS patients was 4.47 years, including 4 cases with the onset of younger than 1 year. Only one patient (5.8%) developed cholecystolithiasis, suggesting that HS patients could benefit from an early diagnosis. MCV, MCH, and MCHC levels in most of HS patients were within normal ranges, which were consistent with previous reports and suggested that conventional laboratory testing was not ideal for the diagnosis of HS (Aggarwal et al., 2020; Wang et al., 2020).

Mutations of the *ANK1* are the most common cause of HS (He et al., 2018; Wu et al., 2021a). Ankyrin-1 protein, encoded by the *ANK1* gene, links *β* spectrin to band 3, and plays a key role in membrane elasticity and mechanical stability. Deficiency of ankyrin-1 leads to decreased spectrin assembly on the membrane (Hanspal et al., 1991; Li et al., 2020). We identified a total of 17 different causative mutations in the *ANK1*, including 15 novel ones and two previously reported. According to the ACMG criteria, 12 novel mutations were considered pathogenic, while the pathogenicity of the other 2 (c.4648A>G and c.5254delTTC) were unknown (Richards et al., 2015).

As of October 2022, 281 mutations of the *ANK1* associated with HS have been reported, including non-sense mutations, missense mutations, splicing mutations, small or gross deletions, insertions, regulatory mutations, and complex rearrangements (https://www.hgmd.cf.ac.uk/ac/all.php). Our data showed that among the 17 mutations of the *ANK1*, there were five non-sense mutations, five frameshift mutations, three splicing mutations, three missense mutations and one in-frame mutation. The three-dimensional (3D) model prediction showed that the protein structure of the 3 novel missense mutations changed, leading to the impaired stability of the erythrocyte membrane structure and erythrocyte destruction.

The genotype-phenotype correlation in HS has been analyzed (Tole et al., 2020; Wu et al., 2021a; Xie et al., 2021a). We compared clinical data in HS patients with different mutation types of the *ANK1*, including RBC, Hb, MCV, MCH, MCHC, RDW-SD, RDW-CV, HCT, Ret%, T-Bil, D-Bil, and LDH. Consistently with previous findings, no significant differences in clinical data were identified in HS patients with different mutation types of *ANK1* probably due to the small sample size (Tole et al., 2020). The MCHC levels in all recruited HS patients were lower than 359 g/L, which was similar to the findings of Wang et al. (2020), further confirming that the conventional laboratory testing of MCHC lacks the specificity and sensitivity.

Structurally, *ANK1* is composed of a N-terminal membrane binding domain, a central spectrin binding domain and a C-terminal regulatory domain. The spectrin proteins (*α* and *β*) and ankyrin are of great significance in maintaining cell membrane stability. In our cases, HS patients with variants of the *ANK1* located in the regulatory domains presented significantly higher Hb levels (97.6 ± 15.63 g/L) compared to those located in the membrane binding domains (73.89 ± 14.22 g/L). Meanwhile, HS patients with variants of the *ANK1* in regulatory domains had lower T-bil levels than those located in membrane binding domains. Our findings were inconsistent with a previous study that patients with mutations of the *ANK1* in the spectrin binding domain present the most severe anemia (van Vuren et al., 2019). However, when we extended the Hb and T-Bil data to all reported HS probands with ANK1 mutations and our cases, only T-Bil levels of HS patients with ANK1 mutations in membrane binding domains seemed higher than those with mutations in spectrin binding domain and regulatory domains, although significant differences were not detectable. Wang et al. (2020) proposed that variants of the *ANK1* death domain, rather than other structural domains, is correlated with low MCV and MCH levels, which was not observed in our study. Correlation analysis between such clinical phenotypes and genotypes need to be conducted in larger samples in the future.

## Conclusion

In summary, we reported 17 unrelated HS children with 17 mutations of the *ANK1*, including 15 novel ones and two previously reported, and analyzed the clinical phenotypes stratified by mutation types and mutation regions. Our study expanded the ANK1 variant spectrum of Chinese people. Genetic testing is a useful tool to predict clinical phenotypes in childhood and guide family counselling of HS, especially in patients without family history.

## Data Availability

The data presented in the study are deposited in the LOVD database with individual ID 00430365, 00430366, and 00430367.
