# Supplementary material for: Clinical manifestations of 17 Chinese children with hereditary spherocytosis caused by novel mutations of the ANK1 gene and phenotypic analysis
Source: Front Genet. 2023 Feb 1;14:1088985. doi: 10.3389/fgene.2023.1088985 (PMC9929461; doi:10.3389/fgene.2023.1088985)
Supplement: Supplementary file 1 [file Table1.DOCX]

Supplementary Table 1. Clinical features of 17 hereditary spherocytosis patients with different types of mutations of the *ANK1* gene in this study

| Clinical data | Nonsense (n=5) | Frameshift (n=5) | Splicing (n=3) | Missense (n=3) | P value |
| --- | --- | --- | --- | --- | --- |
| RBC (×10^12^/L), median (range) | 3.34(2.05-3.71) | 2.41(2.14-3.95) | 2.82(1.8-2.84) | 2.93(2.41-3.1) | 0.81 |
| Hb (g/L), median (range) | 92(53-105) | 70(54-115) | 73(52-82) | 83(70-106) | 0.74 |
| MCV (fL), median (range) | 82.9(79.3-88.3) | 88.4(82.7-91.8) | 83.8(81.2-84.4) | 82.6(82.2-96.8) | 0.28 |
| MCH (pg), median (range) | 27.2(25.9-28.3) | 29(25.2-31.1) | 28.9(25.9-28.9) | 28.3(25.4-34.2) | 0.53 |
| MCHC (g/L), median (range) | 338(308-347) | 329(305-339) | 342(319-345) | 343(308-353) | 0.54 |
| RDW-SD (fL), median (range) | 63.1(55.8-66.1) | 83.6(54.4-94.2) | 61.2(53.2-63.7) | 61.1(55.2-83.6) | 0.27 |
| RDW-CV (%), median (range) | 22.7(20.5-24.9) | 26.5(16.9-35.1) | 20.3(17.7-22.7) | 21.3(15.6-28.6) | 0.35 |
| HCT (fL), median (range) | 26.5(17-30.9) | 21.3(17.7-34.8) | 22.9(15.2-23.8) | 24.2(21.3-30) | 0.79 |
| Ret (%), median (range) | 12.67(7.12-16.7) | 14.77(2.93-21.62) | 9.28(9.2-9.56) | 10.76(8.8-14.77) | 0.40 |
| T-Bil (μmol/L), median (range) | 46.9(33.15-57.1) | 59.24(24.3-86.46) | 53.2(29.3-174.8) | 85.8(24.3-85.8) | 0.79 |
| D-Bil (μmol/L), median (range) | 11.67(8.7-23.9) | 9.7(8.94-12.16) | 16.6(12.4-32) | 11.6(9.3-11.6) | 0.13 |
| LDH (U/L), median (range) | 447(274-541) | 367(248-551) | 331(324-396) | 313(313-551) | 0.79 |

Except for splenectomy and blood transfusion were listed for the case number (n, %), the remaining were expressed as median (range).

RBC, red blood cells; Hb, hemoglobin; MCV, mean corpuscular volume; MCH, mean corpuscular hemoglobin; MCHC, mean corpuscular hemoglobin concentration; RDW-SD, standard deviation of red blood cell distribution width; RDW-CV, coefficient of variation of red blood cell distribution width; HCT, hematocrit; Ret, reticulocyte; T-Bil, total bilirubin; D-Bil, direct bilirubin; LDH, lactate dehydrogenase.

Supplementary Table 2. Clinical features of 17 hereditary spherocytosis with different mutation regions in the *ANK1* gene.

| Clinical data | Membrane binding domain (n=9) | Spectrin binding domain (n=3) | Regulatory domain (n=5) | P value |
| --- | --- | --- | --- | --- |
| RBC (×10^12^/L), median (range) | 2.82(2.06-4.45) | 2.05(1.80-3.54) | 3.34(2.41-3.95) | 0.18 |
| MCV (fL), median (range) | 82.7(62.7-91.8) | 82.9(79.4-84.4) | 88.1(79.3-96.8) | 0.62 |
| MCH (pg), median (range) | 27.2(18.2-31.1) | 26.8(25.9-28.9) | 29(27.5-34.2) | 0.15 |
| MCHC (g/L), median (range) | 315(290-345) | 338(312-342) | 340(329-353) | 0.16 |
| RDW-SD (fL), median (range) | 66.1(39.4-94.2) | 60.4(53.2-63.3) | 55.8(54.4-83.6) | 0.23 |
| RDW-CV (%), median (range) | 22.8(17.2-35.1) | 22.7(17.7-24.9) | 20.5(15.6-26.5) | 0.31 |
| HCT (fL), median (range) | 22.9(17.7-30.1) | 17(15.2-28.1) | 30(21.3-34.8) | 0.09 |
| Ret (%), median (range) | 9.56(1.84-21.62) | 11.94(9.28-14.37) | 13.72(2.93-16.7) | 0.89 |
| D-Bil (μmol/L), median (range) | 14.1(8.94-32) | 11.67(8.70-12.40) | 11.73(9.3-12.7) | 0.70 |
| LDH (U/L), median (range) | 363.5(313-447) | 324(319-468) | 407.5(248-551) | 0.99 |

Supplementary Table 3. Gene mutations and clinical features in Chinese hereditary spherocytosis patients with *ANK1* mutations

| No | Location | DNA change | Effect | Mutation type | Hb (g/l) | Ret (%) | T-bil (μmol/l) | Reference |
| --- | --- | --- | --- | --- | --- | --- | --- | --- |
| 1 | Exon 1 | c.2T>G | p.M1R | start codon mutation | – | – | – | (Peng et al., 2018) |
| 2 | Exon 1 | c.2T>G | p.M1R | start codon mutation | – | – | – | (Wang et al., 2018a) |
| 3 | Exon 1 | c.2T>G | p.M1R | start codon mutation | 54 | 17.71 | 99.7 | (Qin et al., 2020) |
| 4 | Intron 1 | c.28-2A>G | oblig. splice site | Splicing | 104 | – | 52.3 | (Qin et al., 2020) |
| 5 | Exon 3 | c.191T>C | p.L64P | Missense | 74 | – | 177.2 | (Qin et al., 2020) |
| 6 | Exon 4 | c.229-2A>C | oblig. splice site | Splicing | – | – | 118.1 | (Wang et al., 2017a) |
| 7 | Exon 4 | c.T290G | p.L97R | Missense | 60 | 7.57 | 53.53 | (Hao et al., 2019) |
| 8 | Exon 4 | c.319C>T | p.Q107* | Nonsense | 109 | 8.44 | 510.8 | (Wang et al., 2021b) |
| 9 | Exon 6 | c.328+2A>G | oblig. splice site | Splicing | – | – | – | (Peng et al., 2018) |
| 10 | Exon 6 | c.541G > C | p.A181P | Missense | – | – | – | (Peng et al., 2018) |
| 11 | Exon 7 | c.709C>T | p.Q237* | Nonsense | 57 | 8.47 | 395 | (Wang et al., 2021b) |
| 12 | Exon 8 | c.725_728dupCACT | p.H244Tfs*113 | Frameshift | – | – | – | (Wang et al., 2018b) |
| 13 | Exon 8 | c.740C > T | p.S247F | Missense | – | – | – | (Peng et al., 2018) |
| 14 | Exon 8 | c.781C > T | p.R261W | Missense | – | – | – | (Peng et al., 2018) |
| 15 | Exon 8 | c.796G > T | p.E266* | Nonsense | 60.2 | 8.7 | – | (Guan et al., 2018) |
| 16 | Exon 8 | c.810+5G>A | oblig. splice site | Splicing | – | – | – | (Peng et al., 2018) |
| 17 | Exon 9 | c.811G > T | p.D271Y | Missense | – | – | – | (Peng et al., 2018) |
| 18 | Exon 9 | c.824C>G | p.P275R | Missense | 55 | 19 | – | (Xue et al., 2020) |
| 19 | Exon 9 | c.830A>G | p.H277R | Missense | – | – | – | (Zhang and Xu, 2019) |
| 20 | Exon 9 | c.834_833insC | p.C278Wfs*78 | Frameshift | 112 | 5.5 | 521.2 | (Jiang et al., 2016) |
| 21 | Exon 9 | c.841C>T | p.R281* | Nonsense | 48 | 8.55 | 43.4 | (Hao et al., 2019) |
| 22 | Exon 9 | c.841C>T | p.R281* | Nonsense | 80 | 9.32 | 82.1 | (Qin et al., 2020) |
| 23 | Exon 9 | c.856C>T | p.R286* | Nonsense | – | – | – | (Wang et al., 2018b) |
| 24 | Exon 9 | c.856C> T | p.R286* | Nonsense | – | – | – | (Peng et al., 2018) |
| 25 | Exon 9 | c.856C> T | p.R286* | Nonsense | 109 | 11.7 | 148.9 | (Wang et al., 2021b) |
| 26 | Exon 9 | c.858_862delAATCT | p.I287Rfs*67 | Frameshift | 44 | 8.78 | 49.90 | (Gong et al., 2019) |
| 27 | Exon 9 | c.923dupC | p.I309Nfs*47 | Frameshift | 108 | – | 57.8 | (Qin et al., 2020) |
| 28 | Exon 10 | c.985G>C | p.A329P | Missense | – | – | – | (Zhang and Xu, 2019) |
| 29 | Exon 10 | c.990_991delGA | p.E330Dfs*25 | Frameshift | – | – | – | (Peng et al., 2018) |
| 30 | Exon 11 | c.1109dupA | p.N370Kfs*101 | Frameshift | – | – | – | (Wang et al., 2018b) |
| 31 | Intron 12 | c.1305+5G>A | oblig. splice site | Splicing | 73 | – | – | (Qin et al., 2020) |
| 32 | Exon 14 | c.1420delC | p.L474Ffs*13 | Frameshift | 71 | 5.61 | – | (Qin et al., 2020) |
| 33 | Exon 15 | c.1616dupC | p.L540Sfs*81 | Frameshift | 73 | – | – | (Qin et al., 2020) |
| 34 | Exon 16 | c.1717delC | p.L573Cfs*64 | Frameshift | – | – | – | (Peng et al., 2018) |
| 35 | Exon 17 | c.1801-1C>G | oblig. splice site | Splicing | 61 | 11.54 | 86.5 | (Sun et al., 2019) |
| 36 | Exon 17 | c.1814_1818  delCTTTG | p.L606Hfs*13 | Frameshift | – | – | – | (Wang et al., 2018b) |
| 37 | Exon 17 | c.1867C>T | p.Gln623* | Nonsense | – | – | – | (Wang et al., 2018b) |
| 38 | Exon 22 | c.2394_2397 del | p.S799Ifs*5 | Frameshift | 148 | – | 53.4 | (Qin et al., 2020) |
| 39 | Exon 23 | c.2524G> T | p.E842C* | Nonsense | – | – | – | (Peng et al., 2018) |
| 40 | Intron 23 | c.2559-2A>G | oblig. splice site | Splicing | 73 | 11.05 | 78.8 | (Qin et al., 2020) |
| 41 | Intron 25 | c.2735+1G>T | oblig. splice site | Splicing | – | – | – | (Wang et al., 2018b) |
| 42 | Exon 26 | c.2803C>T | p.R935* | Nonsense | – | – | – | (Wang et al., 2018b) |
| 43 | Exon 26 | c.2848A> C | p.T950P | Missense | – | – | – | (Peng et al., 2018) |
| 44 | Exon 26 | c.2891dupC | p.L965Tfs*152 | Frameshift | 85 | 23.57 | 65.8 | (Qin et al., 2020) |
| 45 | Exon 26 | c.2950C>T | p.Q984* | Nonsense | 64 | 12.2 | 38 | (Wang et al., 2021b) |
| 46 | Exon 26 | c.2960+2T>G | oblig. splice site | Splicing | 67 | 3.09 | NA | (Luo et al., 2018) |
| 47 | Intron 27 | c.3115+2T>G | oblig. splice site | Splicing | – | – | – | (Wang et al., 2018b) |
| 48 | Exon 28 | c.3116-1G>C | oblig. splice site | Splicing | – | – | – | (Peng et al., 2018) |
| 49 | Exon 28 | c.3178C> A | p.P1060T | Missense | – | – | – | (Peng et al., 2018) |
| 50 | Exon 28 | c.3179C>T | p.P1060L | Missense | 89 | 17.18 | 93.7 | (Qin et al., 2020) |
| 51 | Exon 28 | c.3275delA | p.Q1092Rfs*13 | Frameshift | – | – | – | (Wang et al., 2018b) |
| 52 | Exon 28 | c.3275delA | p.Q1092Rfs*13 | Frameshift | 62 | 10.90 | 39.60 | (Gong et al., 2019) |
| 53 | Exon 28 | c.3302_3305delinsGAGTGCCGGAGAATGCCG | p.T1101Rfs*9 | Frameshift | – | – | – | (Wang et al., 2018b) |
| 54 | Exon 29 | c.3464G>A | p.W1155* | Nonsense | – | – | – | (Wang et al., 2018b) |
| 55 | Exon 29 | c.3464G>A | p.W1155* | Nonsense | – | – | – | (Peng et al., 2018) |
| 56 | Exon 30 | c.3553T> C | p.W1185R | Missense | – | – | – | (Peng et al., 2018) |
| 57 | Exon 30 | c.3554G>A | p.W1185* | Nonsense | – | – | – | (Wang et al., 2018b) |
| 58 | Exon 31 | c.3754C>T | p.R1252* | Nonsense | 50 | 4.1 | 22 | (Wang et al., 2021b) |
| 59 | Exon 31 | c.3813_3823del | p.Q1272Lfs*100 | Frameshift | 54 | 6.3 | 179 | (Wang et al., 2021b) |
| 60 | Exon 31 | c.3847delA | p.R1283Gfs*3 | Frameshift | 76 | 17.02 | 55.7 | (Wang et al., 2021b) |
| 61 | Exon 33 | c.4000C>T | p.R1334* | Nonsense | – | – | – | (Wang et al., 2018b) |
| 62 | Exon 33 | c.4000C>T | p.R1334* | Nonsense | 79 | 17.88 | 47.5 | (Qin et al., 2020) |
| 63 | Exon 34 | c.4153C>T | p.R1385* | Nonsense | – | – | – | (Wang et al., 2018b) |
| 64 | Exon 34 | c.4276C>T | p.R1426* | Nonsense | 58 | – | – | (Wang et al., 2017b) |
| 65 | Exon 36 | c.4306C>T | p.R1436* | Nonsense | 65 | 9.32 | 100.00 | (Gong et al., 2019) |
| 66 | Exon 36 | c.4306C>T | p.R1436* | Nonsense | – | – | – | (Wang et al., 2018b) |
| 67 | Exon 36 | c.4387_4390delAACA | p.N1463Wfs*17 | Frameshift | – | – | – | (Qin et al., 2020) |
| 68 | Exon 37 | c.4462C>T | p.R1488* | Nonsense | 26 | – | – | (Qin et al., 2020) |
| 69 | Exon 37 | c.4462C>T | p.R1488* | Nonsense | 72 | – | – | (Qin et al., 2020) |
| 70 | Exon 38 | c.5022dupA | p.G1675Rfs*66 | Frameshift | – | – | – | (Peng et al., 2018) |
| 71 | Exon 38 | c.5044C>T | p.R1682* | Nonsense | – | – | – | (Peng et al., 2018) |
| 72 | Exon 40 | c.5422delG | p.E1808Sfs*3 | Frameshift | 40 | 8.2 | 103.7 | (Li et al., 2019) |
| 73 | Exon 40 | c.5455C>T | p.Q1819* | Nonsense | – | – | – | (Wang et al., 2018b) |
| 74 | Intron 36 | c.4391-2 A>C | p.N1463Kfs*4 | Splicing | 65.6 | 6.57 | 27.6 | (Xu et al., 2023) |
| 75 | Exon 22 | c.2394_2397del CAGT | – | Frameshift | 73 | 2.18 | – | (Zhu et al., 2020) |
| 76 | Exon 10 | c.1000delA | p.1334Sfs*6 | Truncated | 69 | 4.6 | 233.6 | (Xie et al., 2021b) |
| 77 | Exon 36 | c.4358_c.4359 delAG | p.E1453Afs*46 | Frameshift | 33 | 9.68 | 36 | (Wu et al., 2021) |
| 78 | Exon 22 | c.2489_c.2492 delTAGT | p.L830Sfs*7 | Frameshift | 73 | 13.27 | 20.1 | (Wu et al., 2021) |
| 79 | Exon 34 | c.4123C > T | p.R1375X,523 | Truncated | 67 | 17.11 | 46.5 | (Wu et al., 2021) |
| 80 | – | c.2395-2(IVS20) A > G | (NM_ 001142446) | Splicing | 73 | 17.31 | 51.7 | (Wu et al., 2021) |
| 81 | Exon 35 | c.4276C > T | p.R1426X,472 | Truncated | 65 | 5.88 | 111.8 | (Wu et al., 2021) |
| 82 | Exon 2 | c.226C > T | p.Q76X,1822 | Truncated | 62 | 5.12 | 19.9 | (Wu et al., 2021) |
| 83 | Exon 16 | c.1858_c.1859delCT | p.L620Afs*33 | Frameshift | 70 | 12.04 | 13.5 | (Wu et al., 2021) |
| 84 | Exon 35 | c.4276C > T | p.R1426X,472 | Truncated | 43 | 13.41 | 88.8 | (Wu et al., 2021) |
| 85 | Exon 9 | c.955C > T | p.R319X,1579 | Truncated | 40 | 14.3 | 102.2 | (Wu et al., 2021) |
| 86 | – | c.1504-9(IVS13) G > A | (NM_001142446) | Mutations near the shear site | 55 | 14.13 | 44.4 | (Wu et al., 2021) |
| 87 | Exon 8 | c.790C > T | p. Gln264Ter | Nonsense | 112 | – | 194.33 | (Chai et al., 2020) |
| 88 | Exon 25 | c.2693dupC | p.A899Sfs*11 | Frameshift | 80 | 4.8 | 32.5 | (Huang et al., 2019) |
| 89 | – | c.3392delT | p.Leu1131Argfs*15 | Frameshift | 109 | 39 | – | (Li et al., 2022) |
| 90 | Exon 23 | c.G2467T | p.E823X | Nonsense | 62 | 11.2 | 22.67 | (Fu et al., 2022) |
| 91 | Exon 9 | c.856C > T | p.Arg286Ter | Nonsense | 120 | – | – | (Wang et al., 2020) |
| 92 | Intron 16 | c.1800 + 1G > A | – | Splicing | – | – | – | (Wang et al., 2020) |
| 93 | Intron 23 | c.2559-2A > C | – | Splicing | – | – | – | (Wang et al., 2020) |
| 94 | Exon 7 | c.701delT | p.Phe234SerfsTer19 | Indel | 119 | – | – | (Wang et al., 2020) |
| 95 | Exon 1 | c.2T > A | p.Met1Lys | start-loss | 159 | – | – | (Wang et al., 2020) |
| 96 | Exon 10 | c.1032_1034delGGC | p.Ala346del | Indel | 84 | – | – | (Wang et al., 2020) |
| 97 | Exon 8 | c.735delC | p.Ile245MetfsTer8 | Indel | 86 | – | – | (Wang et al., 2020) |
| 98 | Exon 37 | c.4414C > T | p.Gln1472Ter | Nonsense | 74 | – | – | (Wang et al., 2020) |
| 99 | Exon 1 | c.2T > A | p.Met1Lys | start-loss | 87 | – | – | (Wang et al., 2020) |
| 100 | Exon 32 | c.3865delG | p.Glu1289LysfsTer16 | Indel | 78 | – | – | (Wang et al., 2020) |
| 101 | Exon 5 | c.341C > T | p.Pro114Leu | Missense | 92 | – | – | (Wang et al., 2020) |
| 102 | Exon 23 | c.2531_2532insT | p.Asp845GlyfsTer24 | Indel | 88 | – | – | (Wang et al., 2020) |
| 103 | Exon 35 | c.4253G > A | p.Trp1418Ter | Nonsense | 85 | – | – | (Wang et al., 2020) |
| 104 | – | c.4707G>A | p.Trp1569* | Nonsense | 139 | 8.94 | 96.1 | (Zhu et al., 2023) |
| 105 | Exon 4 | c.399Thr>Gly | p.Tyr133X,1765 | Nonsense | – | – | – | (Xie et al., 2021a) |
| 106 | Exon 17 | c.1914_1918delTTTGC | p.Pro638Profs*14 | Frameshift | – | – | – | (Xie et al., 2021a) |
| 107 | Exon 14 | c.1564delC | p.Leu522Cysfs*44 | Frameshift | – | – | – | (Xie et al., 2021a) |
| 108 | Exon 37 | c.4439dupA | p.Asn1480Kfs*20 | Frameshift | – | – | – | (Xie et al., 2021a) |
| 109 | Exon 37 | c.4510_4513del | p.Asn1504Trpfs*17 | Frameshift | – | – | – | (Xie et al., 2021a) |
| 110 | Exon 27 | c.2961delC | p.T988P fs*30 | Frameshift | – | – | – | (Xie et al., 2021a) |
| 111 | Exon 18 | c.2142dupT | p.Pro715Serfs*111 | Frameshift | – | – | – | (Xie et al., 2021a) |
| 112 | Intron 26 | c.2858+1G>C | – | Splicing | – | – | – | (Xie et al., 2021a) |
| 113 | Exon 28 | c.3235delG | p.Glu1079fs | Frameshift | – | – | – | (Xie et al., 2021a) |
| 114 | Exon 39 | c.4739A>G | p.Gln1580Arg | Missense | – | – | – | (Xie et al., 2021a) |
| 115 | Exon 25 | c.2638‐2 A>G | – | Splicing | – | – | – | (Xie et al., 2021a) |
| 116 | Exon 27 | c.2926C>T | p.Arg976Ter | Nonsense | – | – | – | (Xie et al., 2021a) |
| 117 | Exon 34 | c.4153C>T | p.Arg1385X | Nonsense | – | – | – | (Xie et al., 2021a) |
| 118 | – | c.841C > T | p.Arg281Ter | Nonsense | 132 | 12.18 | 413.9 | (Wang et al., 2021c) |
| 119 | – | c.1941C>A | p.His647Gln | Missense | 60 | 15.3 | 312.5 | (Zhao et al., 2022) |
| 120 | Exon 27 | c.3051G>A | p.W1017X | Nonsense | 65 | 3.6 | – | (Xiang and Shen, 2022) |
| 121 | Exon 33 | c.4086delC | p.M1363fs*43 | Frameshift | 60 | – | – | (Xiang and Shen, 2022) |
| 122 | Exon 6 | c.558delG | p.R187Afs*66 | Frameshift | 65 | 18.4 | – | (Xiang and Shen, 2022) |
| 123 | Exon 29 | c.3280C>T | p.R1094X | Nonsense | 64 | 0.9 | – | (Xiang and Shen, 2022) |
| 124 | Exon 9 | c.909+1_909+2insG | – | Splicing | 66 | 3 | 37.87 | (Wang et al., 2021a) |
| 125 | Exon 37 | c.G4393T | p.E1465X | Nonsense | 58 | 5 | 53.92 | (Wang et al., 2021a) |
| 126 | Exon 9 | c.940C>T | p.R314X | Nonsense | 73 | 10 | 65.85 | (Wang et al., 2021a) |
| 127 | – | c.955C>T | p.Arg319X | Nonsense | 77 | 13.8 | 63.4 | (Bin et al., 2020) |
| 128 | Exon 23 | c.2462-2A>G | – | Splicing | 73 | 9.56 | 174.8 | This study |
| 129 | Exon 19 | c.2164C>T | p. Q722X | Nonsense | 56 | 7.12 | 55.7 | This study |
| 130 | Exon 2 | c.2393_2394insTAGT | p. D800Qfs*1 | Frameshift | 102 | 13.89 | 86.46 | This study |
| 131 | Exon 14 | c.1564delC | p. Q522Afs*12 | Frameshift | 64 | 18.49 | – | This study |
| 132 | Exon 17 | c.1801-2A>G | – | Splicing | 82 | 9.2 | 53.2 | This study |
| 133 | Exon 22 | c.2461G>A | p. G821R | Missense | 70 | 12.72 | 85.8 | This study |
| 134 | Exon 39 | c.5163_5173del | p. W1721Cfs*16 | Frameshift | 115 | 2.93 | 37.38 | This study |
| 135 | Exon 37 | c.4399_4400insGA | p. Q1467Sfs*16 | Frameshift | 70 | 14.77 | 24.3 | This study |
| 136 | Exon 38 | c.4648A>G | p. I1550V | Missense | 106 | – | – | This study |
| 137 | Exon 39 | c.5299G>T | p. E1767X | Nonsense | 105 | 12.67 | 46.9 | This study |
| 138 | Exon 30 | c.3533-1G>A | – | Splicing | 52 | 9.28 | 29.3 | This study |
| 139 | Exon 1 | c.52_54delTTC | p. F18del | In frame | 81 | 1.84 | – | This study |
| 140 | Exon 37 | c.4414C>T | p. Q1472X | Nonsense | 92 | 16.7 | 34.1 | This study |
| 141 | Exon 9 | c.872T>G | p. L291R | Missense | 83 | 8.8 | – | This study |
| 142 | Exon 30 | c.3600C>A | p. C1200X | Nonsense | 53 | 14.37 | 57.1 | This study |
| 143 | Exon 31 | c.3754C>T | p. R1252X | Nonsense | 95 | 11.94 | 33.15 | This study |
| 144 | Exon 22 | c.2390_2393del | p. L797Sfs*7 | Frameshift | 54 | 21.62 | 81.1 | This study |

The variants shown in this Table were described using the NM_000037.4 for ANK1 transcript reference sequence.

Hb, hemoglobin; Ret, reticulocyte; T-Bil, total bilirubin

**Reference:**

Bin, Q., Wang, D., and Li, X. (2020). A case report of ANK1 gene Arg319X mutation in hereditary spherocytosis. *J China Pediatr Blood Cancer* 25(2), 98-100. doi: 10.3969/j.issn.1673-5323.2020.02.010.

Chai, S., Jiao, R., Sun, X., Fu, P., Zhao, Q., and Sang, M. (2020). Novel nonsense mutation p. Gln264Ter in the ANK1 confirms causative role for hereditary spherocytosis: a case report. *BMC Med Genet* 21(1), 223. doi: 10.1186/s12881-020-01161-4.

Fu, P., Jiao, Y., Chen, K., Shao, J., Liao, X., Yang, J., et al. (2022). Targeted next-generation sequencing identifies a novel nonsense mutation in ANK1 for hereditary spherocytosis: A case report. *World J Clin Cases* 10(15), 4923-4928. doi: 10.12998/wjcc.v10.i15.4923.

Gong, J., He, X., Zou, R., Chen, K., You, Y., Zou, H., et al. (2019). [Clinical characteristics and genetic analysis of hereditary spherocytosis caused by mutations of ANK1 and SPTB genes]. *Zhongguo Dang Dai Er Ke Za Zhi* 21(4), 370-374. doi: 10.7499/j.issn.1008-8830.2019.04.013.

Guan, H., Liang, X., Zhang, R., Wang, H., Liu, W., Zhang, R., et al. (2018). Identification of a de novo ANK1 mutation in a Chinese family with hereditary spherocytosis. *Hematology* 23(6), 357-361. doi: 10.1080/10245332.2017.1398210.

Hao, L., Li, S., Ma, D., Chen, S., Zhang, B., Xiao, D., et al. (2019). Two novel ANK1 loss-of-function mutations in Chinese families with hereditary spherocytosis. *J Cell Mol Med* 23(6), 4454-4463. doi: 10.1111/jcmm.14343.

Huang, T., Sang, B., Lei, Q., Song, C., Lin, Y., Lv, Y., et al. (2019). A de novo ANK1 mutation associated to hereditary spherocytosis: a case report. *BMC Pediatr* 19(1), 62. doi: 10.1186/s12887-019-1436-4.

Jiang, M., Lu, J., Zhong, Y., Wang, Y., and Yang, C. (2016). [Identification of a novel ANK1 gene mutation in a newborn with hereditary spherocytosis]. *Zhonghua Yi Xue Yi Chuan Xue Za Zhi* 33(1), 44-47. doi: 10.3760/cma.j.issn.1003-9406.2016.01.011.

Li, D., Li, B., Li, S., Li, W., Wang, Y., and Guo, X. (2019). [Analysis of ANK1 gene mutation in a family with hereditary spherocytosis type I]. *Zhonghua Yi Xue Yi Chuan Xue Za Zhi* 36(10), 999-1001. doi: 10.3760/cma.j.issn.1003-9406.2019.10.012.

Li, J., Guo, H., Zhu, Z., and Sun, J. (2022). A Novel ANK1 Mutation in a Neonatal Hereditary Spherocytosis Case: Diagnostic Challenges and Familial Genetic Analysis. *Acta Haematol* 145(6), 575-581. doi: 10.1159/000525054.

Luo, Y., Li, Z., Huang, L., Tian, J., Xiong, M., and Yang, Z. (2018). Spectrum of Ankyrin Mutations in Hereditary Spherocytosis: A Case Report and Review of the Literature. *Acta Haematol* 140(2), 77-86. doi: 10.1159/000492024.

Peng, G., Yang, W., Zhao, X., Jin, L., Zhang, L., Zhou, K., et al. (2018). [The characteristic of hereditary spherocytosis related gene mutation in 37 Chinese hereditary spherocytisis patients]. *Zhonghua Xue Ye Xue Za Zhi* 39(11), 898-903. doi: 10.3760/cma.j.issn.0253-2727.2018.11.005.

Qin, L., Nie, Y., Zhang, H., Chen, L., Zhang, D., Lin, Y., et al. (2020). Identification of new mutations in patients with hereditary spherocytosis by next-generation sequencing. *J Hum Genet* 65(4), 427-434. doi: 10.1038/s10038-020-0724-z.

Sun, Q., Xie, Y., Wu, P., Li, S., Hua, Y., Lu, X., et al. (2019). ANK1Targeted next-generation sequencing identified a novel mutation associated with hereditary spherocytosis in a Chinese family. *Hematology* 24(1), 583-587. doi: 10.1080/16078454.2019.1650873.

Wang, C., Zhang, J., Zhan, Z., and Liang, X. (2021a). Gene and family analysis of 3 cases of hereditary spherocytosis. *Guizhou Yi Yao* 45(9). doi: 10.3969/j.issn.1000-744X.2021.09.042.

Wang, D., Song, L., Shen, L., Zhang, K., Lv, Y., Gao, M., et al. (2021b). Mutational Characteristics of Causative Genes in Chinese Hereditary Spherocytosis Patients: a Report on Fourteen Cases and a Review of the Literature. *Front Pharmacol* 12, 644352. doi: 10.3389/fphar.2021.644352.

Wang, J., Ma, L., Gong, X., Cai, C., and Sun, J. (2021c). de novoSevere hyperbilirubinemia in a neonate with hereditary spherocytosis due to a ankyrin mutation: A case report. *World J Clin Cases* 9(19), 5245-5251. doi: 10.12998/wjcc.v9.i19.5245.

Wang, R., Yang, S., Xu, M., Huang, J., Liu, H., Gu, W., et al. (2018a). Exome sequencing confirms molecular diagnoses in 38 Chinese families with hereditary spherocytosis. *Sci China Life Sci* 61(8), 947-953. doi: 10.1007/s11427-017-9232-6.

Wang, R., Yang, S., Xu, M., Huang, J., Liu, H., Gu, W., et al. (2018b). Exome sequencing confirms molecular diagnoses in 38 Chinese families with hereditary spherocytosis. *Sci China Life Sci* 61(8), 947-953. doi: 10.1007/s11427-017-9232-6.

Wang, X., Mao, L., Shen, N., Peng, J., Zhu, Y., Hu, Q., et al. (2017a). ANK1An IVS3-2A>C mutation causes exon 4 skipping in two patients from a Chinese family with hereditary spherocytosis. *Oncotarget* 8(68), 113282-113286. doi: 10.18632/oncotarget.22936.

Wang, X., Yi, B., Mu, K., Shen, N., Zhu, Y., Hu, Q., et al. (2017b). de novoIdentification of a novel ANK1 R1426* nonsense mutation in a Chinese family with hereditary spherocytosis by NGS. *Oncotarget* 8(57), 96791-96797. doi: 10.18632/oncotarget.18243.

Wang, X., Zhang, A., Huang, M., Chen, L., Hu, Q., Lu, Y., et al. (2020). Genetic and Clinical Characteristics of Patients With Hereditary Spherocytosis in Hubei Province of China. *Front Genet* 11, 953. doi: 10.3389/fgene.2020.00953.

Wu, C., Xiong, T., Xu, Z., Zhan, C., Chen, F., Ye, Y., et al. (2021). Preliminary Study on the Clinical and Genetic Characteristics of Hereditary Spherocytosis in 15 Chinese Children. *Front Genet* 12, 652376. doi: 10.3389/fgene.2021.652376.

Xiang, Y., and Shen, Y. (2022). Report of seven new mutation sites in hereditary spherocytosis and review of the literature. *J Mod Med Health* 38(2), 357-360. doi: 10.3969/j.issn.1009-5519.2022.02.049.

Xie, F., Lei, L., Cai, B., Gan, L., Gao, Y., Liu, X., et al. (2021a). Clinical manifestation and phenotypic analysis of novel gene mutation in 28 Chinese children with hereditary spherocytosis. *Mol Genet Genomic Med* 9(4), e1577. doi: 10.1002/mgg3.1577.

Xie, L., Xing, Z., Li, C., Liu, S., and Wen, F. (2021b). Identification of a De Novoc.1000delA ANK1 mutation associated to hereditary spherocytosis in a neonate with Coombs-negative hemolytic jaundice-case reports and review of the literature. *BMC Med Genomics* 14(1), 77. doi: 10.1186/s12920-021-00912-3.

Xu, L., Wei, X., Liang, G., Zhu, D., Zhang, Y., Zhang, Y., et al. (2023). A novel splicing mutation of ANK1 is associated with phenotypic heterogeneity of hereditary spherocytosis in a Chinese family. *Biochim Biophys Acta Mol Basis Dis* 1869(1), 166595. doi: 10.1016/j.bbadis.2022.166595.

Xue, J., He, Q., Xie, X., Su, A., and Cao, S. (2020). A clinical and experimental study of adult hereditary spherocytosis in the Chinese population. *Kaohsiung J Med Sci* 36(7), 552-560. doi: 10.1002/kjm2.12198.

Zhang, Y., and Xu, Z. (2019). Clinical and genetic features of children with hereditary spherocytosis: an analysis of 4 cases. *Zhongguo Dang Dai Er Ke Za Zhi* 21(1), 29-32. doi: 10.7499/j.issn.1008-8830.2019.01.006.

Zhao, J., Hong, F., Lu, S., Zhu, M., and Xu, M. (2022). A new mutation of ANK1 gene in hereditary spherocytosis: case report. *ZheJiang Yi Xue* 44(18), 2000-2002. doi: 10.12056/j.issn.1006-2785.

Zhu, F., Liang, M., Xu, L., Peng, Z., Cai, D., Wei, X., et al. (2020). A tetranucleotide deletion in the ANK1 gene causes hereditary spherocytosis; a case of misdiagnosis. *Gene* 726, 144226. doi: 10.1016/j.gene.2019.144226.

Zhu, X., Peng, M., Yin, Y., Zhang, Y., Zheng, D., Peng, Z., et al. (2023). Identification of a novel ANK1 mutation in a Chinese family with hereditary spherocytosis: A case report. *Exp Ther Med* 25(1), 4. doi: 10.3892/etm.2022.11704.
